# The Influence of *Chlorella* and Its Hot Water Extract Supplementation on Quality of Life in Patients with Breast Cancer

**DOI:** 10.1155/2014/704619

**Published:** 2014-03-02

**Authors:** Naoto Noguchi, Isao Maruyama, Akira Yamada

**Affiliations:** ^1^Research Laboratory, Chlorella Industry Co., Ltd., 1343 Hisatomi, Chikugo, Fukuoka 833-0056, Japan; ^2^Research Center for Innovative Cancer Therapy, Kurume University, Kurume 830-0011, Japan

## Abstract

A self-control, randomized, and open-label clinical trial was performed to test the effects of the unicellular green algae *Chlorella* and hot water extract supplementation on quality of life (QOL) in patients with breast cancer. Forty-five female patients with breast cancer who were living at home and not hospitalized were randomly assigned to 3 groups receiving vitamin mix tablet (control), *Chlorella* granules (test food-1), or *Chlorella* extract drink (test food-2) daily for one month. The Functional Assessment of Cancer Therapy-Breast (FACT-B), the Izumo scale for abdominal symptom-specific QOL, and a narrative-form questionnaire were used to determine outcomes. Data of thirty-six subjects were included for final analysis. FACT-B scores at presupplementation found no significant group differences in all subscales. Scores on the breast cancer subscale in the *Chlorella* granule group significantly increased during the supplementation period (*P* = 0.042). Fifty percent of the *Chlorella* extract group reported positive effects by the test food such as reduction of fatigue and improvements of dry skin (*P* < 0.01 versus control group). The findings suggested the beneficial effects of *Chlorella* on breast cancer-related QOL and of *Chlorella* extract on vitality status in breast cancer patients. These findings need to be confirmed in a larger study.

## 1. Introduction

Complementary and alternative medicine (CAM) is widely used by patients who are undergoing or have completed medical treatment for cancer [1, 2]. While conventional therapies, including surgery, radiation therapies, and anticancer drugs, are targeted at tumors, their side effects on normal organs may significantly compromise quality of life (QOL) during and after treatment. For many patients, CAM approaches may be pursued in order to augment the conventional modalities' anticancer effects, as well as to reduce treatment-related symptoms and other side effects that diminish QOL [2, 3]. CAM therapies include medication therapies (natural products involving the use of herbs, animal parts, and/or minerals) and nonmedication therapies carried out primarily without the use of medication (such as acupuncture or manual therapy) [2]. Since many kinds of natural products such as herbs, special foods, mushrooms, and shark cartilage are already being used by many cancer patients, research into their safety and efficacy for cancer patients is an urgent issue [2, 4].

*Chlorella* is a genus of unicellular green algae that reproduce at a rapid rate and has been used as a food source and nutritional supplement for over half a century [5, 6]. At present, *Chlorella* is widely used as nutritional supplement by healthy persons as well as chronic disease invalids and cancer patients. *Chlorella* contains a high amount of protein and dietary fibers, as well as many kinds of vitamins and essential minerals. It is also rich in plant pigments, including chlorophylls, lutein, and *β*-carotene. Many health-promoting benefits, such as the lowering of oxidative stress and serum lipid and the excretion of toxic substances including dioxin and methyl mercury, have been reported in both animals and humans [7–13]. Hot water extract of *Chlorella* contains a high amount of polysaccharides, including galactose, mannose, rhamnose, ribose, and xylose [14], and has been reported to improve host resistance to viral infection, bacterial infection, and tumors in animals [15–19]. Recently, we confirmed that hot water extract of *Chlorella* improves the perforin and granulysin preservation ratio in NK cells and killer T-cells in healthy subjects by a placebo-controlled double-blind study (unpublished data). *Chlorella* supplementation has also been shown to improve outcomes in several chronic diseases in humans [10, 20].

Breast cancer is the most common malignant disease affecting women of all ages [21]. A majority of breast cancer patients have used some kind of natural products as CAM [2, 4]. Due to the documented benefits of *Chlorella* and its hot water extract in the QOL of chronic disease invalids and in immunocompetence and host resistance to tumors in animals, we studied the effects of *Chlorella* granules and hot water extract supplementation on QOL in patients with breast cancer.

## 2. Methods

### 2.1. Subjects, Study Design, and Test Food

Female patients with breast cancer were recruited from the membership of a patient support nonprofit organization, Wig Ring Japan (Fukuoka, Japan). All subjects were breast cancer survivors who had completed curative treatment (surgery and/or radiotherapy) or were undergoing or had undergone chemotherapy and/or hormone therapy; all were within 24 months of the end of the medical treatment. We excluded patients who (1) were pregnant or lactating, (2) had severe underlying diseases other than breast cancer, (3) were taking warfarin, other alternative medicines, or supplements, and (4) had allergies to the test foods. Subjects were asked to immediately withdraw from this study when the attending doctor recommended the discontinuance of the experimental food. The present study was conducted according to the guidelines laid down in the Declaration of Helsinki, and all procedures involving human subjects were approved by the Ethics Committee of the Chlorella Industry (ethics number K-103). All participants gave their written informed consent prior to study initiation.

**Figure 1:**
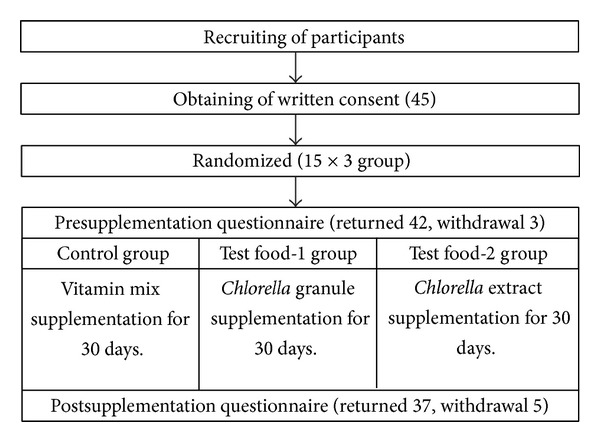
The study design flowchart and subject numbers at various stages.

This was self-control, randomized, and open-label clinical trial. The study design flowchart and subject numbers at various stages are shown in Figure 1. Forty-five subjects who submitted a written consent were randomly assigned to three treatment groups: vitamin mix, *Chlorella* granule, or *Chlorella* extract. Subject groups of 15 were established in anticipation of some withdrawal, according to the recommendation of sample size of 12 per group for a pilot study [22]. Subjects daily received vitamin mix tablets as a control, *Chlorella *granules as test food-1, or *Chlorella* extract drink as test food-2 for 30 days. Three subjects withdrew before the start of the examination because they changed their minds about participation. Five subjects withdrew from the study during the supplementation period, two because of progression of their disease, one who felt mentally burdened by the daily intake of test food, and two because they changed their minds about participation. One subject was excluded due to poor compliance (65%) with test food unrelated to side effects or changes in general health status. Accordingly, data of thirty-six subjects were included in the final data analysis; their average compliance with test food consumption was 95.6% with a confidence interval at the 95% level of 93.0–98.3%. This study was performed from February 2012 to May 2012.

The control supplement was “Nature Made B Complex” tablet (vitamin mix, Otsuka Pharmaceutical Co., Ltd., Tokyo, Japan). One tablet, which contains 15 mg of vitamin B_1_, 12 mg of vitamin B_2_, 10 mg of vitamin B_6_, 15 *μ*g of vitamin B_12_, 10 mg of niacin, 10 mg of pantothenic acid, 200 *μ*g of folic acid, and 30 *μ*g of biotin, was daily administered after breakfast. Test food-1 was “Biorinck Granule” (*Chlorella* granules, Chlorella Industry Co., Ltd., Tokyo, Japan). Four sticks were daily administered as follows: 2 sticks after breakfast, 1 stick after lunch, and 1 stick after dinner. Four sticks of *Chlorella* granules contain 6000 mg of *Chlorella* dry powder (*Parachlorella beijerinckii*). Test food-2 was “Biorinck BCEx 503” drink (*Chlorella* extract, Chlorella Industry Co., Ltd., Tokyo, Japan). One bottle of the drink was administered in the morning, afternoon, and night, for a total of 3 bottles daily of 2400 mg (as polysaccharides) of *Chlorella* hot water extract.

### 2.2. Questionnaires

Self-completed questionnaires, the Functional Assessment of Cancer Therapy-Breast (FACT-B, version 4) scale, and the Izumo scale for abdominal symptom-specific QOL were given to all subjects at the pre- and postsupplementation period. The FACT-B includes a 36-item questionnaire divided into five subscales: physical well-being (7 items), social/familial well-being (7 items), emotional well-being (6 items), functional well-being (7 items), and breast cancer subscale (9 items) [23, 24]. These subscales comprise QOL-related statements that respondents rate on a 5-point Likert scale of agreement ranging from “not at all” (score 0) to “very much” (score 4). Item scores within a subscale were summed to produce a subscale score. The five subscale scores were then summed to obtain the total FACT-B score. A higher score indicates a more favorable QOL. The Izumo scale is an abdominal symptom-specific QOL questionnaire that includes 15 items in 5 domains—reflux, pain, fullness, constipation, and diarrhea, with 3 items in each domain [25]. Questions were rated on a 6-point Likert scale, from “not at all” (score 0) to intolerable (score 5). The scores of the three questions within a domain were added to determine domain-specific scores. A lower score indicates a more favorable abdominal symptom-specific QOL. At presupplementation, an additional questionnaire inquiring about demographics and medical treatment characteristics was used. At postsupplementation, an additional questionnaire inquiring about compliance (frequency of test food intake and remaining test food supply), medical treatment during supplementation period, favorable effect of test food on physical condition (yes/no and how), and detrimental effect of test food on physical condition (yes/no and how) was used.

### 2.3. Statistical Analysis

Scores of FACT-B and Izumo scales were expressed as medians, 25th percentiles, and 75th percentiles. Kruskal-Wallis test was used to compare data among the three groups. For comparison of pre- and postsupplementation values in each treatment group, Wilcoxon signed-ranks test was used. The *χ* ^2^-test was used to compare enumerated data among the three groups. If a statistically significant difference (*P* < 0.05) was detected, Fisher's exact test was performed for comparison between the control group and one of the two test groups. Demographic data, age, and months after the end of medical treatment were expressed as means and standard deviations. One-way ANOVA was used to compare means among the three groups.

## 3. Results

Demographic data and medical histories are shown in Table 1. The 36 subjects were all women. Their mean age was 50.8 ± 10.9 years. All were living at home and not hospitalized, 83% of them (30/36) were married, and 44% of them (16/36) had a job (full time or part time). Most subjects (89%, 32/36) had undergone surgery, mastectomy, or breast-conserving surgery. Thirty-one percent of subjects (11/36) had completed radiotherapy. Thirty-nine percent (14/36) had completed chemotherapy. Thirty-one percent (11/36) were undergoing chemotherapy and continued it during the experimental period. Forty-four percent (16/36) continued hormone therapy during the experimental period. If the period after medical treatment was estimated as zero months in the case of undergoing chemotherapy, the mean period after the end of the curative treatment (surgery, radiotherapy, and/or chemotherapy) was 6.6 ± 6.4 months. For all demographic data and medical histories, there were no significant differences among the three groups (Table 1).

Changes in the FACT-B score during the supplementation period are shown in Table 2. There were no significant differences among the three groups in the five subscale scores and total scores at the beginning of the experimental period. In both the vitamin mix group and *Chlorella* extract group, there were no significant differences between pre- and postsupplementation for all five subscale scores and total scores. On the other hand, breast cancer subscale scores of the *Chlorella* granule group significantly increased at postsupplementation compared with presupplementation (*P* < 0.05). The breast cancer subscale can be used to assess breast cancer-related QOL and includes 9 questions regarding symptoms due to the disease or treatment such as shortness of breath and hair loss, and psychological aspects related to the disease and treatment such as worry over the effect of stress on the illness and feeling like a woman. When breast cancer subscale's scores of *Chlorella* granule group were evaluated for each individual item, two items scores significantly increased at postsupplementation compared with presupplementation (*P* < 0.05): the score of “One or both of my arms are swollen or tender” was increased from 3.0 (1.0, 3.5) to 3.0 (2.0, 4.0) and that of “I worry that other members of my family might someday get the same illness I have” was increased from 1.0 (0.0, 2.0) to 2.0 (1.5, 3.0). Two other items scores showed a tendency of increase (*P* < 0.1): the score of “I am bothered by hair loss” increased from 1.5 (0.3, 3.8) to 3.5 (3.0, 4.0) and that of “I worry about the effect of stress on my illness” increased from 1.0 (0.0, 1.0) to 2.0 (0.5, 3.0). The increase of these items scores was considered to contribute to the improvement of the breast cancer subscale in the *Chlorella* granule group.

Changes in Izumo scale scores during the supplementation period are shown in Table 3. Subjects that had some abdominal symptoms at presupplementation were limited, with 49% (17/35) having reflux, 43% (15/35) pain, 57% (20/35) fullness, 68% (23/34) constipation, and 37% (13/35) diarrhea. There were no significant differences among the three groups in all of the 5-domain scores. There were also no significant differences between pre- and postsupplementation in all the 5-domain scores.

Favorable effects of test foods on physical conditions are shown in Table 4. No subjects in the vitamin control group (0/13) reported positive effects, whereas 1 (1/11; 9%) in the *Chlorella* granule group and fully 50% (6/12) of the *Chlorella* extract group did. The difference among the three groups was significant (*P* = 0.004), as was that between the *Chlorella* extract group and the control (*P* < 0.01). The favorable effect in the *Chlorella *granule group was reduction of fatigue. Those in the *Chlorella* extract group included reduction of fatigue (2), improvement of abdominal symptoms (2), improvements of dry skin (2), improvement of hair gloss (1), and improvements of cold constitution (1). Detrimental effects were reported for 1/13 (constipation and diarrhea) in the vitamin mix group and 1/11 (constipation) in the *Chlorella* granule group. However, these symptoms were slight and transitory, so they did not hinder the continuation of test food supplementation. None of the subjects in the *Chlorella* extract group reported any detrimental effects.

## 4. Discussion

This study was the first trial to assess the effects of supplementation with *Chlorella *and its hot water extracts on the QOL of patients with breast cancer. The findings showed the possibility of therapeutic effects of *Chlorella* granules and *Chlorella* extract on the QOL. Rising stress levels make it very difficult for cancer patients to care for themselves, resulting in psychological and physical problems and higher health care costs. Yet, the evidence has suggested that a significant reduction in managing of disease- and treatment-related symptoms could improve the QOL among women with cancer [26]. Thus, *Chlorella* treatment has a potentially important role to play in managing psychological distress in cancer patients. This study also suggests that *Chlorella* and its hot water extract can be used safely with breast cancer.

In the FACT-B questionnaire, the breast cancer subscale score significantly increased after 30 days of *Chlorella* granule supplementation (*P* < 0.05). This improvement in the breast cancer subscale depended on significant improvements in the scores for the items “One or both of my arms are swollen or tender” and “I worry that other members of my family might someday get the same illness I have.” Many studies have shown the effects of *Chlorella* on oxidative stress lowering in both animals and humans, because of the abundance of antioxidants including lutein, *β*-carotene, and vitamin E [7, 8, 27]. Accordingly, oxidative stress lowering and anti-inflammatory actions by the supplementation of *Chlorella* might contribute to improving of the item score of “One or both of my arms are swollen or tender,” through the improvement of lymph flow and lymphedema. QOL of breast cancer patients has been known to recover gradually after completion of curative treatment such as surgery and chemotherapy [28, 29]. In this study, there was a significantly positive correlation between the score of the breast cancer subscale at presupplementation and the period (months) after the end of medical treatment (*y* = 0.36*x* + 17.89, *P* < 0.05, data not shown). There were no significant correlations between other subscale scores and the period after the end of medical treatment. These results suggested that the recovery of QOL after medical treatment tended to reflect on the FACT-B breast cancer subscale in our cohort.

In the Izumo scale questionnaire for abdominal symptom-specific QOL, there were no significant differences in any domain score between pre- and postsupplementation and among the 3 groups. Although abdominal symptoms are a usual side effect of chemotherapy, the number of subjects having abdominal symptoms at presupplementation was limited. Assessment of test food effects on abdominal QOL might therefore be difficult in our cohort.

Subjects who experienced positive effects by *Chlorella* extract reached 50%, significantly more than the controls (*P* < 0.01). The positive effects reported included reduction of fatigue, improvement of abdominal symptoms, improvements of dry skin, improvement of hair gloss, and improvement of cold constitution. As mentioned above, *Chlorella* extract has been shown to improve host resistance to viral and bacterial infection and tumors [15–19]. The successes of *Chlorella* extract in improving the QOL of cancer patients can be explained by its favorable effects on immune functions, since augmentation of immunity improves the health-related QOL including vitality status and bowel movements [30]. When the test foods of *Chlorella* granules and *Chlorella* extract were estimated together, subjects who experienced positive effects of test foods were 30%, which is comparable to the previous report in which positive effects were experienced by 24.3% of CAM users with cancer in Japan [2]. When the group of subjects who reported positive effects of test food by narrative-form questionnaire was examined using the FACT-B score, no significant improvement was observed. These results suggest that narrative-form questionnaire is useful for obtaining evidence about patients' subjective experience during the test period.

Although the current study adopted a specially designed vitality questionnaire to evaluate the effects of *Chlorella* and its hot water extract, the absence of a placebo-control group leaves open the possibility that its positive findings are attributable to the placebo effect of test foods. Since *Chlorella *is becoming popular as a health food among patients with chronic diseases and cancers, it is possible that its effect on the QOL of some subjects was exaggerated because they placed high confidence in its effectiveness. Although the possibility of a placebo effect cannot be entirely ruled out, the setting of the randomized control group and good compliance rates might suggest the generalizability of the findings. The documented benefits of *Chlorella* and its hot water extract in QOL in patients with chronic diseases [10, 20], in oxidative stress lowering [7, 8, 27], and in modulation of immune responses and enhancing antitumor immunity [15–19] also support the finding. This study, a self-control, randomized, and open-label clinical design, is the first step toward exploring the efficacy of *Chlorella* and its hot water extract on QOL in breast cancer subjects. Results from outcome studies could provide evidence for its efficacy and might be used to design future comprehensive studies.

## 5. Conclusions

This is the first study of the effects of *Chlorella* and its hot water extract supplementation on quality of life in a population of patients with breast cancer. Results suggested that the supplementation with *Chlorella* improves breast cancer-related QOL as evaluated by the FACT-B questionnaire. Half of the patients who took *Chlorella* extract experienced positive effects on their physical condition such as reduction of fatigue and improvement of dry skin. These findings need to be confirmed in larger studies.

## Figures and Tables

**Table 1:** Demographic data and medical histories.

	Vitamin mix	*Chlorella* granule	*Chlorella* extract	*P* value^a^
	(*n* = 13)	(*n* = 11)		
Age	51.2 ± 10.9	50.5 ± 14.0	50.8 ± 8.5	0.985
Marital status				
Married	12	9	9	0.504
Others	1	2	3	
Occupation status				
Having a job	6	3	7	0.322
Having no job	7	8	5	
Anticancer treatment				
Mastectomy	8	7	6	0.769
Breast-conserving surgery	4	3	4	0.951
Radiotherapy	4	2	5	0.474
Chemotherapy (completed)	7	5	2	0.141
Chemotherapy (undergoing)	3	4	4	0.755
Undergoing hormone therapy	6	4	6	0.796
Months after treatment^b^	7.1 ± 5.5	5.2 ± 6.0	7.3 ± 8.0	0.708

^a^Among 3 groups. ^b^Months after the end of curative treatment (surgery, radiotherapy, and/or chemotherapy). In case of undergoing chemotherapy, period after the treatment was estimated as 0 months.

**Table 2:** Changes in the FACT-B scores during the supplementation period.

	*n*	Presupplementation	Postsupplementation	*P* value^a^
Physical well-being				
Vitamin mix	12	25.0 (21.5, 26.0)	26.0 (23.0, 27.0)	0.139
*Chlorella* granule	10	20.5 (11.3, 24.5)	25.0 (18.8, 27.5)	0.107
*Chlorella* extract	12	21.5 (18.0, 25.3)	23.0 (17.0, 26.9)	0.790
Social/familial well-being				
Vitamin mix	13	16.3 (12.8, 22.0)	16.8 (14.0, 25.0)	0.093
*Chlorella* granule	10	20.8 (15.8, 22.8)	21.5 (19.0, 22.1)	0.359
*Chlorella* extract	12	21.0 (16.8, 22.5)	16.9 (9.8, 22.0)	0.169
Emotional well-being				
Vitamin mix	12	19.0 (11.8, 20.5)	19.5 (13.5, 20.5)	0.386
*Chlorella* granule	10	17.0 (13.0, 19.8)	18.0 (13.3, 20.8)	0.722
*Chlorella* extract	11	18.0 (15.0, 19.1)	17.0 (15.5, 20.5)	0.310
Functional well-being				
Vitamin mix	12	21.0 (17.3, 24.5)	22.0 (19.3, 25.8)	0.294
*Chlorella* granule	10	21.0 (16.0, 23.3)	20.5 (17.0, 22.5)	0.834
*Chlorella* extract	11	19.0 (14.5, 26.5)	22.0 (13.0, 25.5)	0.563
Breast cancer subscale				
Vitamin mix	12	21.5 (20.0, 24.5)	20.5 (17.8, 27.1)	0.965
*Chlorella* granule	11	16.0 (12.9, 21.5)	20.4 (17.5, 22.5)	0.042**∗**
*Chlorella* extract	11	23.0 (19.5, 23.5)	20.0 (18.0, 23.0)	0.478
Total				
Vitamin mix	12	102.4 (89.5, 113.8)	104.6 (91.0, 117.2)	0.099
*Chlorella* granule	10	91.2 (72.8, 100.5)	104.6 (90.9, 110.2)	0.241
*Chlorella* extract	11	94.0 (87.0, 118.3)	91.0 (80.5, 112.4)	0.328

Median (25th percentile, 75th percentile).^ a^Between pre- and postsupplementation. *Statistically significant.

**Table 3:** Changes in Izumo scale scores during the supplementation period.

	*n*	Presupplementation	Postsupplementation	*P* value^a^
Reflux				
Vitamin mix	13	0.0 (0.0, 1.0)	0.0 (0.0, 1.0)	0.753
*Chlorella* granule	11	1.0 (0.0, 6.5)	1.0 (0.0, 6.0)	0.237
*Chlorella* extract	11	2.0 (0.0, 3.5)	2.0 (0.0, 4.0)	0.554
Pain				
Vitamin mix	13	0.0 (0.0, 1.0)	0.0 (0.0, 3.0)	0.889
*Chlorella* granule	11	1.0 (0.0, 4.0)	0.0 (0.0, 4.0)	0.361
*Chlorella* extract	11	0.0 (0.0, 1.5)	0.0 (0.0, 2.5)	0.173
Fullness				
Vitamin mix	13	0.0 (0.0, 3.0)	0.0 (0.0, 2.0)	0.735
*Chlorella* granule	11	1.0 (0.0, 5.5)	1.0 (0.0, 3.0)	0.178
*Chlorella* extract	11	2.0 (0.5, 3.5)	2.0 (0.0, 3.5)	0.463
Constipation				
Vitamin mix	13	3.0 (0.0, 5.0)	1.0 (0.0, 3.0)	0.161
*Chlorella* granule	10	4.0 (0.5, 7.0)	2.5 (0.3, 3.0)	0.310
*Chlorella* extract	11	3.0 (0.0, 4.0)	2.0 (0.5, 4.5)	1.000
Diarrhea				
Vitamin mix	13	0.0 (0.0, 3.0)	0.0 (0.0, 2.0)	0.686
*Chlorella* granule	11	2.0 (0.0, 3.0)	3.0 (0.0, 4.5)	0.091
*Chlorella* extract	11	0.0 (0.0, 0.0)	0.0 (0.0, 3.0)	0.273

Median (25th percentile, 75th percentile).^ a^Between pre- and postsupplementation.

**Table 4:** Favorable effects of test foods on physical conditions.

	Vitamin mix	*Chlorella* granule	* Chlorella* extract	*P* value^a^
Yes	0% (0/13)	9% (1/11)	**50% (6/12)**	0.004
Reasons		Reduction of fatigue	Reduction of reflux, improvements of dry skin and hair gloss, improvement of bowel movement, improvement of cold constitution, reduction of fatigue, and reduction of fatigue and improvement of dry skin.	

^a^Among 3 groups. **P < 0.01 versus vitamin mix.
